# Mother–infant interaction in schizophrenia: transmitting risk or resilience? A systematic review of the literature

**DOI:** 10.1007/s00127-015-1127-x

**Published:** 2015-10-10

**Authors:** Kirstine Agnete Davidsen, Susanne Harder, Angus MacBeth, Jenna-Marie Lundy, Andrew Gumley

**Affiliations:** Department of Psychology, University of Southern Denmark, Campusvej 55, 5230 Odense, Denmark; Department of Child and Adolescent Mental Health Odense, Research Unit, Mental Health Services in the Region of Southern Denmark, Odense, Denmark; Department of Psychology, University of Copenhagen, Copenhagen, Denmark; School of Health in Social Science, University of Edinburgh, Edinburgh, Scotland, UK; Institute of Health and Wellbeing, University of Glasgow, Glasgow, Scotland, UK

**Keywords:** Mother–infant interaction, Schizophrenia, Resilience, psychological, Risk factors, Transmission

## Abstract

**Purpose:**

The parent–infant relationship is an important context for identifying very early risk and resilience factors and targets for the development of preventative interventions. The aim of this study was to systematically review studies investigating the early caregiver–infant relationship and attachment in offspring of parents with schizophrenia.

**Methods:**

We searched computerized databases for relevant articles investigating the relationship between early caregiver–infant relationship and outcomes for offspring of a caregiver with a diagnosis of schizophrenia. Studies were assessed for risk of bias.

**Results:**

We identified 27 studies derived from 10 cohorts, comprising 208 women diagnosed with schizophrenia, 71 with other psychoses, 203 women with depression, 59 women with mania/bipolar disorder, 40 with personality disorder, 8 with unspecified mental disorders and 119 non-psychiatric controls. There was some evidence to support disturbances in maternal behaviour amongst those with a diagnosis of schizophrenia and there was more limited evidence of disturbances in infant behaviour and mutuality of interaction.

**Conclusions:**

Further research should investigate both sources of resilience and risk in the development of offspring of parents with a diagnosis of schizophrenia and psychosis. Given the lack of specificity observed in this review, these studies should also include maternal affective disorders including depressive and bipolar disorders.

**Electronic supplementary material:**

The online version of this article (doi:10.1007/s00127-015-1127-x) contains supplementary material, which is available to authorized users.

## Introduction

Children of parents with schizophrenia are at increased risk of developing psychiatric disorder compared to the general population. Having one parent with schizophrenia results in 7 % lifetime risk of schizophrenia [1] and 55 % risk of developing any psychiatric condition [2]. Children of parents with schizophrenia display motor-cognitive delay [3], emotional problems during preschool, attention difficulties and poorer social adjustment at school [4]. High-risk studies [5–7] identify interactions between genetic factors, obstetric complications and neurodevelopment in the transmission of risk during the antenatal and perinatal periods [8, 9]. Recent studies emphasize that environmental and psychosocial variables including social adversity [10], urban/inner city living [11], migration and ethnicity [12] also play an important role in understanding pathways towards schizophrenia [13]. Childhood adversity and trauma are linked to increased risk of psychosis [14], with emerging evidence for the role of stress sensitivity as an underlying biological substrate [15].

A small number of high-risk studies have examined the early care-giving environment, finding that experiencing prolonged institutional care and parental separation were linked to the development of schizophrenia compared to other diagnostic groups [16]. In their meta-analysis, de Sousa and colleagues [17] showed that parental communication deviance is robustly associated with offspring psychosis. There is increasing evidence to suggest that people with schizophrenia are more likely to display insecure (particularly avoidant) attachment patterns, which are associated with poorer outcomes including poorer engagement with services, more frequent and longer hospitalization, greater trauma and more positive and negative symptoms [18].

These attachment studies rely on retrospective evaluations of the early care-giving, providing no prospective empirical data on experiences and characteristics of early parental relationships, despite evidence of the clinical and theoretical importance of the early care-giving environment as a basis for the emergence of risk and resilience as it materializes in later life. Although there have been developmental psychopathology informed conceptual reviews of the schizophrenia literature [19], there has been no systematical survey of the literature on the early care-giving environment in schizophrenia. We sought to address this via the following questions:

1. What are the characteristics of the studies investigating the early caregiver–infant relationship?
2. What are the characteristics of the early caregiver–infant relationship and what are its correlates?
3. What methodological features are associated with increased risk of bias?

## Methods

### Inclusion and exclusion criteria

All studies were cohort or case–control studies with either cross-sectional or longitudinal outcomes and included (i) participants who were caregivers with a diagnosis of schizophrenia; (ii) participants also included infants and young children between the ages of 0 and 6 years; (iii) reported data on caregiver–infant interaction; (iv) were published between 1968 and November 2013; and (v) were written in English. Excluded studies were (i) qualitative methods; (ii) case studies; (iii) dissertations; and (iv) conference abstracts.

### Search strategy

A PRISMA systematic review was conducted by searching PsycINFO, PubMed and Google Scholar computerized databases. Search terms used the following combined Thesaurus and MeSH terms: [“MOTHERS”] and [“PSYCHOSIS” or “SCHIZOPHRENIA”] and [“INFANT” or “CHILDREN” or “OFFSPRING”] and [“ATTACHMENT” or “INTERACTION” or “RELATION*”]. Online titles and abstracts were reviewed after de-duplication. Articles not meeting inclusion criteria were discarded. Full texts of potentially eligible articles were obtained. Reference lists of eligible articles were searched to identify relevant articles that may have been missed by the electronic search strategy. Two additional cohorts were identified [20, 21].

### Risk of bias

We systematically assessed the risk of bias via a methodological evaluation of all studies (SH & AG) using methods developed for observational studies in epidemiology [22]. We assessed the following methodological domains: Selection, Measures, Loss to Follow-up, Blinding of Outcomes, Confounding, and Statistical Methods. The Risk of Bias is summarized in Table 1. Overall agreement was calculated as Kappa = 0.76. Where differences were identified, these were resolved through discussion.

**Table 1:** Risk of Bias

	Methods for selecting study participants	Methods for measuring exposure and outcome variables	Design-specific sources of bias (excluding confounding) loss to follow-up	Design-specific sources of bias (excluding confounding) blinding of outcomes	Methods to control confounding	Statistical methods (excluding control of confounding)
Lund Cohort [23–34]	Low	High	High	Unclear	Low	High
Rochester Cohort [35, 36]	Unclear	High	High	Unclear	Low	High
Emory Cohort [20, 37]	High	Low	High	Low	Low	Low
Pittsburgh Cohort [38, 39]	High	Low	N/A	Low	High	High
Boston Cohort [40]	High	Low	N/A	Low	High	Low
Bethlem Cohort [41]	High	High	High	High	High	Low
Austin Cohort [21]	High	High	Low	High	High	High
Manchester Cohort 1993-1995 [42]	High	High	N/A	High	High	High
Manchester Cohort 1993–1995 and 1996-2000 [43]	High	Low	N/A	Low	Low	Low
Manchester Cohort 1996–2000 [44, 45]	High	Low	N/A	Low	Unclear	Low
London Cohort I (Pawlby) [46]	High	Low	High	Low	High	Low
London Cohort II (Kenny) [47]	High	Low	Low	Low	High	Low

## Results

**Fig. 1:**
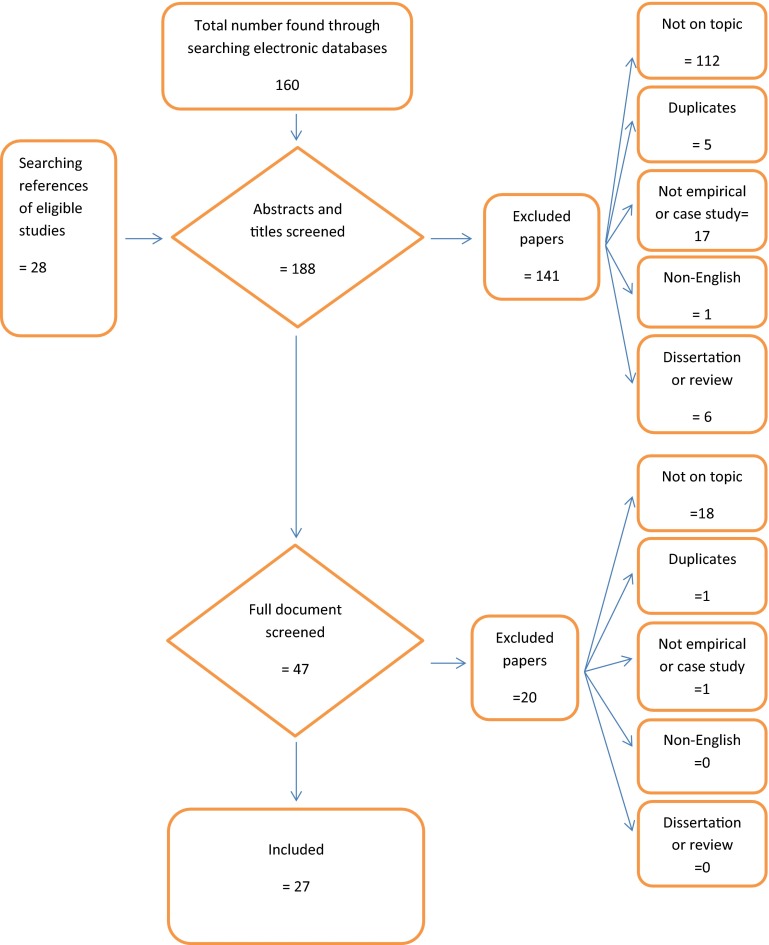
Flow chart of systematic search and review process

The search process is summarized in Fig. 1. We identified 160 potentially eligible papers, and a further 28 from references. 141 papers were excluded on the basis of the abstracts and titles alone. We screened full manuscripts for 47 studies. We excluded 20 after three reviewers (KD, AG & SH) scrutinized the manuscripts. A fourth independent blind reviewer (JML) replicated the search process. No new studies were identified.

### What are the characteristics of the studies investigating the early caregiver–infant relationship?

We included a total of 27 papers representing *k* = 10 cohorts (See Table 2). These studies comprised women diagnosed with schizophrenia (*n* = 208), other psychoses (*n* = 27), depression (*n* = 203), mania/bipolar disorder (*n* = 59), personality disorder (*n* = 40), unspecified mental disorders (*n* = 8) and non-psychiatric controls (*n* = 119). Amongst those with psychosis/schizophrenia, median age was 28.6 years (range 21.0–34.6). For the infants, median age was 8.3 months (range 3 days to 14.2 months). Studies were classified into three categories: longitudinal cohorts, cross-sectional cohorts and mother–baby unit studies.

**Table 2:** Characteristics of included studies of mother–infant interaction in schz

Cohort, collection period	Reference study	Design and sample characteristics		Maternal diagnosis and subgroups (mean age)	Infant age*, gender: male (%)	Domains covered				
						Maternal behaviour	Infant behaviour	Mutual interaction	Mother correlates	Infant correlates
		Design	*N*, consent rate, attrition							
Lund Cohort 1973–1977	[23] Study description, no interactive data presented	Longitudi-nal 0–6 year	192, consent rate index: 93 % control 98 %	104 matched controls 88 psychosis (28.7 years) Hereof 17 schz project criteria +RDC	0–6 years Schz: 11(65 %) Psychosis: 50 (57 %) Control: 48 (46 %)					
	[24]		124	51 psychosis, hereof 11 schz 73 matched controls	3 days 58 (47 %)	**√**	**√**	**√**	Diagnosis	
	[25]		3 weeks: 102 6 weeks: 129	42 psychosis, hereof 11 schz 60 matched controls 51 psychosis, hereof 14 schz 78 matched controls	3 and 6 weeks gender: NI	**√**	**√**	**√**	Diagnosis	
	[26]		3½ month:128 6 months: 131	48 psychosis, hereof 11 schz 80 controls 52 psychosis 14 schz 79 controls	3½ and 6 month gender: NI	**√**	**√**	**√**	Diagnosis	
	[27]		126	46 psychosis, hereof 10 schz 80 controls	1 year gender: NI	**√**	**√**	**√**	Diagnosis	Attachment
	[28]		126	46 psychosis, hereof 10 schz 80 controls	1 year 61 (48 %)		**√**		Diagnosis Psychiatric condition	Attachment
	[29]		126	46 psychosis 80 controls	1 year gender: NI	**√**	**√**	**√**	Diagnosis	Attachment
	[30]		126	46 psychosis, hereof 10 schz 80 controls	1 year 61 (48 %)		**√**		Psychiatric condition	Gender
	[31]		Subsample: 126	46 psychosis, hereof 10 schz 80 controls	1 year male: 61 (48 %)		**√**		Diagnosis	
	[32]		126	46 psychosis 80 controls	1 year gender: NI	**√**	**√**	**√**		Fear of strangers
	[33]		NI	88 psychosis hereof 17 with post-partum psychosis					Diagnosis Post-partum Psychosis	Fear of stranger Attachment
	[34]		161 attrition: psych 27 % control 6 %	88 psychosis, hereof 12 schz 104 controls	1 year (attachment) 6 years (psychopathology) gender: NI		**√**			
Roche-ster Cohort 1970–1976	[35] Study description, no interactive data presented		184 attrition 0 to 4 months: 22 %. 4 months to 4 years: 19 %	Matched sample: 29 schz 58 depr 40 Pers. Disorder 57 No mental disorder DSM II criteria	0, 4, 12, 30 and 48 months gender: NI				Diagnosis Severity and chronicity of illness Social status	Mental and psychomotor development
	[36]	0–30 months longitudinal study	184 attrition: whole group: 31 % Schz group: 35 %	Matched sample (24.4 years): 29 schz 58 depr 40 Pers. Disorder 57 No mental disorder DSM II criteria	4 and 12 months gender: NI	**√**	**√** Incl attachment		Diagnosis Illness Severity and chronicity Social status	Mental and psychomotor development
Emory Cohorts	[20]	Longitudinal	153 attrition 29 %	115 index hereof 71 schz 36 depr 8 unspec 38 control DSM III criteria	0–5 year mean: 2 years gender: NI	**√**	**√**	**√**	Diagnosis	IQ Social behaviour Psychiatric outcome
	[37]		101	53 schz 25 depr 23 matched control	3 month to 5 years mean 2 years gender: 50/50	**√**	**√**	**√**	Diagnosis	IQ Social behaviour
Pitts-burgh Cohort	[38] Study description, no interactive data presented	Cross sectional	21	9 schz 12 controls	3 months	**√**	**√**	**√**	Diagnosis Level of anxiety, depression and anger Nurturant care	IQ Neurological and physiological examination
	[39]	Cross-sectional	18	9 schz (21 years) 9 controls (22 years)	Mean 14.2 months gender: NI	**√**	**√**	**√**	Diagnosis Education Care	Mental and psychomotor development
Boston Cohort	[40]	Cross-sectional	45	15 schz 15 depr 15 controls DSMIII criteria	Mean 12.5 months gender: NI		**√**		Diagnosis	
Beth-lem Cohort	[41]	Pre-post. assessment	78	15 schz (26.8 years) 28 depr (29.7 years) 35 bipolar/manic (28.2 years) RDC	Mean age: schz: 5.1, depr: 6.9, bipolar: 4.3, 3 weeks male: schz: 7 (41 %) depr: 17 (60 %) bipolar:18 (51 %)	**√**			Diagnosis	
Austin Cohort	[21]	Pre-post assessment	15	15 schz (28.6 years). DSM-IV criteria	1–44 weeks mean: 16.9 weeks male: 8 (53)	**√**	**√**	**√**	Severity of illness	
Man-chester 1993–95 Cohort	[42]	Cross sec-tional	48 admitted 32 eligible 5 refused to be videotaped 26 included	8 schz (31.5 years) 12 depr 6 bipolar (25.7 years) RDC = research diagnostic criteria	App. 4 months 13 (50 %)	**√**	**√**	**√**	Diagnosis	
Man-chester 1993–95 + 1996–2000 Cohort	[43]	Cross sec-tional replication study	55 included 38 used for analysis (>4 months old from 1993 cohort excluded)	13 Schz 14 bipolar 11 depr ICD-10 diagnostic criteria	4 months gender: NI	**√**	**√**	**√**	Diagnosis	
Man-chester 1996–2000	[44]		45	14 schz 31 affective (28 years) DSM-IV criteria	4–60 weeks, mean: 15 weeks 20 (44 %)	**√**	**√**		Diagnosis	
	[45]		51	14 schz Comparison group: 8 bipolar, 25 depr., 2 OCD, 1 anxiety (28 years) ICD-10 criteria	7–60 weeks, mean: 16 weeks 25 (50 %)	**√**			Diagnosis	
London Cohort	[46]	Pre-post assessment	139 49 control 82 + 8 eligible in-patients	49 controls Of 42 remaining in-patients: 15 schz (34.6 years) 23 depr (32.2 years) 12 mania (29.0 years) DSM-IV criteria	0-52 weeks mean age: At admission: 10.6 weeks At discharge: 19.1 weeks	**√**	**√**		Diagnosis	
	[47]	Pre-post assessment	49	8 schz 23 depr 18 mania	1–53 weeks (admission) 5–61 weeks (discharge) mean age: At admission:12.4 At discharge: 20.6	**√**	**√**		Diagnosis	

Abbreviations: NI = no information, Schz = Schizophrenia, Depr = Depression, RDC = Research Diagnostic Criteria

* Studies reporting on a broad age group are entered according to mean age

#### Longitudinal cohorts

There were three longitudinal cohorts. The Lund Cohort [23] described a study of offspring from 0–6 years comprising 192 participants (*n* = 88 psychosis; *n* = 17 Schizophrenia). The Rochester Cohort [36] described a 0–30 months follow-up of 184 participants (*n* = 29 schizophrenia). The Emory Cohort [20] described a study of offspring from 0–5 years, following up 153 women (*n* = 71 schizophrenia).

#### Cross-sectional cohorts

There were two cross-sectional cohorts. The Pittsburgh Cohort [39] with 18 participants (n = 9 schizophrenia), and the Boston Cohort [40] with 45 participants (*n* = 15 schizophrenia).

#### Mother baby unit cohorts

There were five cohorts derived from consecutive admissions to Mother Baby Units (MBU). The Bethlem Cohort [41] comprised 78 women (*n* = 15 schizophrenia). The Austin Cohort [21] comprised 15 women all of whom met DSM-IV criteria for schizophrenia. The Manchester Cohort (1993–1995) [42] comprised 48 women (*n* = 8 schizophrenia). The Manchester Cohort (1996–2000) [44] comprised 45 women with DSM-IV/ICD-10 diagnoses (*n* = 14 schizophrenia). The London Cohort [46] comprised 42 participants (*n* = 15 schizophrenia).

### Characteristics of the early caregiver–infant relationship and correlates

#### Neonatal

Two studies [24, 41] reported neonatal interaction data. One [24] found atypical maternal behaviour (less maternal social contact during feeding) exhibited by women diagnosed with schizophrenia, in comparison to matched normal controls. For the broader psychosis group, there were also significantly higher levels of maternal tension and uncertainty. Infants of mothers with psychosis showed lower levels of engagement and social contact. Hipwell & Kumar [41] reported nurses’ observations of mother–infant interaction at three time points during admission to an MBU. At each time point, mothers diagnosed with schizophrenia were observed to have significantly higher disturbed behaviour compared to the depressed and bipolar controls. Irrespective of diagnostic group, maternal–infant interaction improved over time. Maternal diagnosis was the only variable predicting greater likelihood of mother–infant dyads being placed on the at-risk register or recommendation for social services supervision/foster care rather than being discharged home unsupervised.

Additional data on studies exploring maternal behaviour, infant behaviour and their interaction amongst neonates are given in Table 3 (Online Resource 1).

#### 1–12 months (maternal behaviour)

Data on maternal behaviour were reported in 13 studies from 7 cohorts [21, 25–27, 29, 32, 36, 42–47]. All but the last two reported associations between maternal diagnosis of schizophrenia and atypical maternal behaviour in interaction. In most studies, the interaction sequence was a 5–30 min unstructured or semi-structured mother–infant play situation. All studies from the Lund Cohort also coded a feeding situation, one study reported from 2 h non-specified home observation [36] and one study [41] based interaction ratings on an MBU.

In the Lund Cohort, maternal schizophrenia and psychosis were associated with reduced social contact during feeding and bodily contact during play, and greater tension/uncertainty at 6 weeks [25]. At 6 months, maternal schizophrenia and psychosis were only associated with reduced social contact during play [26]. By 1 year, maternal schizophrenia and psychosis were associated with increased tension/uncertainty during feeding [27]. Most variables were not linked to secure versus insecure attachment amongst infants at 12 months within the maternal psychosis group. However, greater maternal tension/uncertainty at 12 months was associated with insecure attachment amongst infants [29]. In addition, greater maternal tension/uncertainty at 6 weeks, 3½ months and 12 months was associated with an absence of Fear of Strangers (FoS) amongst offspring of mothers with psychosis [32]. Reduced FoS in infants is consistent with insecure-avoidant and insecure-disorganized attachment patterns.

The Rochester Cohort [36] found that maternal schizophrenia was associated with reduced spontaneity and proximity at 4 but not 12 months. Impairments in a range of maternal behaviours were consistently associated with social status not diagnosis. Snellen and colleagues [21] found that maternal schizophrenia was associated with reduced eye, physical and vocal contact.

Maternal schizophrenia was compared to affective controls in four studies [42–45]. Differences were found at 4 months [42, 43], and within the first year [44, 45]. Maternal schizophrenia was associated with being more remote, silent, verbally and behaviourally intrusive, self-absorbed, flaccid, insensitive, unresponsive, less demanding, displaying less emotional warmth and acceptance and engaging in less infant-focused speech. Pawlby et al. [46] found no differences between maternal schizophrenia and affective controls for maternal mind-mindedness, and no effect of schizophrenia/depression/mania diagnosis on amount of change during admission [47].

#### 1–12 months (infant behaviour)

Infant behaviour was reported in 16 studies from seven cohorts [21, 26–32, 36, 40, 42–44, 46, 47]. Infant behaviours were coded from the same interaction situations as the coding of maternal behaviour (above) and the Strange Situation Procedure [48].

McNeil and colleagues [26] found evidence of reduced social contact at 3.5 months. Two studies using overlapping samples from the two Manchester cohorts found that infants of mothers diagnosed with schizophrenia were less attentive to the mother at 4 months, less engaged with environment and less lively compared to affective controls [42, 43]. Infant attentiveness was associated with maternal sensitivity and responsiveness. Infants who were less attentive were interacting with mothers who were more avoidant, less engaged in the environment and less lively during interactions [43]. Compared to normal controls, infants of mothers diagnosed with schizophrenia were found to be insecurely attached at 12 months [28, 40]. In comparison to maternal depression, offspring of mothers with schizophrenia were found to be more avoidant where offspring of mothers with depression were more ambivalent [40]. Finally, one study found reduced FoS (12 months) in infants with mothers with schizophrenia [30]. Seven studies found no significant differences in infant behaviour compared to matched normal controls at 3 and 6 weeks, or 4, 6 and 12 months [25–27, 31, 36, 44, 46].

#### 1–12 months (mutual interaction)

Mutual engagement during mother–infant interaction was assessed in 8 studies from 4 cohorts [21, 25–27, 29, 32, 42, 43].

Compared to normal controls, two studies did not find significant differences in harmonious interaction for offspring of maternal schizophrenia at 3 weeks, 3½-months and 6 months [25, 26] although significantly less harmonious interaction was noted at 6 weeks [25]. No significant differences were observed at 1 year comparing maternal schizophrenia or psychosis and their offspring to normal controls [27]. Compared to affective controls, maternal schizophrenia and their infants were observed to have less mutually satisfying, engaged, smooth and easy interaction at 4 months [25, 42, 43].

Amongst the dyads, insecure attachment at 12 months was associated with less harmonious feeding at 3 weeks and 12 months and less reciprocity at 6 months [29]. This suggests that early indicators of disturbances in harmony and reciprocity are linked to the emergence of insecure attachment in this group. Consistent with this, Persson-Blennow and colleagues [32] showed that reduced FoS was associated with less harmonious interaction during feeding at 6 months. Finally, Snellen and colleagues [21] showed that mutuality of attention, reciprocity, synchronicity and intensity of interaction all improved during admission to an MBU.

Additional data on maternal behaviour, infant behaviour and mutual interaction from 1- to 12-months are given in Table 4 (Online Resource 1).

In summary, the majority of studies investigating the time period between 1 and 12 months found some evidence for disturbed maternal behaviour in schizophrenia, although the findings were inconsistent over time. Effects appeared to be more consistent for the broader category of maternal psychosis [23] suggesting that some of the inconsistent effects observed in the narrower maternal schizophrenia comparisons may be artifacts of poor statistical power. Evidence that infant behaviour amongst offspring of maternal schizophrenia or maternal psychosis differed from normal controls was more equivocal when coding attachment security during the Strange Situation Procedure. Compared to normal controls, there was greater insecurity and avoidance in offspring of mothers with schizophrenia. Consistent with this avoidant stance, reduced FoS was observed amongst offspring of mothers with schizophrenia. FoS and attachment insecurity were associated with reduced mutually harmonious interactions early in the course of development up to 6 months. Finally, there was less optimal mutuality of interaction amongst offspring and mothers with schizophrenia although this was not consistent across all time points across the first 1−12 months.

#### 13–36 months (maternal behaviour)

There were limited and conflicting data pertaining to maternal behaviour between 13 and 36 months. Three studies from two cohorts [20, 37, 39], reported data on maternal behaviour in mother–infant interaction. In two studies, data were based on a semi-structured play situation and observation in home [20, 37]. Both studies found that, compared to normal controls, maternal behaviour with their children (mean age 2-years, range 0–5 years) in the schizophrenia group was associated with reduced responsiveness and stimulation [20], less affectionate involvement and poorer child-rearing environment [37]. In contrast, Schachter et al. [39] did not find any differences between maternal schizophrenia and normal controls with respect to positive affectionate behaviour, negative angry behaviour or attention during a laboratory feeding task although this study only had 9 participants in each group.

#### 13–36 months (infant behaviour)

Three studies from two cohorts [20, 37, 39] reported data on infant behaviour. Goodman [20] explored infant behaviour during play in the home environment. They found that infants of maternal schizophrenia and depression expressed less affect during play. In addition, children of maternal schizophrenia showed reduced anger and anxiety, reduced communicative competence, increased activity, reduced expression of affection and69. annoyance, less use of mother as a resource and less role play. Goodman & Brumley [37] found no differences for children of maternal schizophrenia or depression compared to normal controls. Maternal affectional involvement was associated with children’s IQ and social competence. Schachter et al. [39] found no differences between children of maternal schizophrenia and normal controls.

#### 13–36 months (mutual interaction)

Two studies from two cohorts [37, 39] reported data on mutual interaction. Schachter et al. [39] found that compared to normal controls, mothers with a diagnosis of schizophrenia showed higher contingency towards their child’s behaviour. Goodman and Brumley [37] found that compared to normal controls, the affective quality of the interaction between children and mothers with schizophrenia was lower and characterized by less anger and hostility.

Additional data from studies exploring maternal behaviour, infant behaviour and mutual interaction amongst children aged between 13 and 16 months are summarized in Table 5 (Online Resource 1).

In summary, there are limited data for associations between maternal behaviour, infant behaviour and mutual interaction amongst cohorts of children aged between 13 and 36 months. The Shachter et al. study [39] is notably underpowered and utilized an invalidated coding system. The *Emory Cohort* [20] described follow-up of 153 women (*n* = 71 schizophrenia; *n* = 36 depression; *n* = 8 unspecified and *n* = 38 non-psychiatric controls). This study found some evidence of differences in maternal behaviour, infant behaviour and mutuality of interaction.

#### 36 months and above

Only the Lund Cohort reported data beyond 36 months [34], reporting longitudinal associations at 6 years. They found that severity of child psychopathology was higher in offspring of maternal schizophrenia and psychosis compared to normal controls. The study found that association of psychopathology and earlier ratings of attachment security at 1 year was not significant.

Additional data on the study beyond 36 months are given in Table 6 (Online Resource 1).

### What methodological features are associated with increased risk of bias?

#### Sampling and design

Of the 10 cohorts included in this systematic review, only three were longitudinal follow-up studies [20, 26, 36]. This means that there were limited data describing the unfolding developmental processes linked to maternal and infant behaviour and their interaction. There were four cohorts where rates of participation and consent were clearly reported [41–43, 46]. These four cohorts were based on consecutive admissions to an MBU. Across the 10 cohorts, there were 208 women diagnosed with schizophrenia and 71 with other psychoses. Most studies were based on small samples. Variance between studies was large and methods of diagnosis varied encompassing DSM-II, III & IV, Research Diagnostic Criteria and ICD 10 criteria as well as study-specific diagnostic criteria. We noted the frequent use of statistical analyses without adjustment for multiple testing.

#### Assessment of mother–infant behaviour and their interaction

The quality of assessment tools for assessing mother–infant behaviour varied between studies. The Emory Cohort [20] used the Mothers Project Rating Scales of Mother–child interaction [50], which has been found to discriminate between emotionally disturbed and well women. High reliability was reported. The Manchester Cohort (1993–1995) [42] and the Manchester Cohort (1996–2000) [43–45] used the Global Rating Scales of Mother–Infant Interaction which have been demonstrated to have good validity and have been used in a number of previous studies. These studies also reported good inter-rater reliability. The Manchester Cohort (1996–2000) [44] also used a modified version of the Stanley et al. classification system [51] for assigning deviant communication in a community sample of depressed mothers. Reliability was reported and there was good agreement for infant behaviour. Agreement for maternal behaviour was moderate. These measures did find significant differences between groups in the studies included in this review.

Two cohorts [21, 41] utilized the Bethlem Mother–Infant Interaction Rating Scale (BMIS) which has been demonstrated to show good psychometric properties. The two studies using the BMIS did not report inter-rater reliability. In contrast, The Lund Cohort [23], The Rochester Cohort [36] and the Pittsburg Cohort [39] used their own methods developed within the study to assess mother–infant behaviour and interaction. The London Cohort [46] coded maternal mind-mindedness using a coding scheme developed for assessing mother–infant interaction in psychologically healthy mothers as well as a non-standardized coding scheme for assessing maternal responsivity. No significant differences were identified.

#### Attachment

The Strange Situation Procedure (SSP) was used in three cohorts [28, 36, 40]. Two studies used abbreviated procedure using three or four of the eight episodes in the Strange situation procedure [28, 36]. The third study, which used the full Strange Situation Procedure, found the largest proportion on insecure attachment in the schizophrenia group [40]. Only two-way (secure insecure) and three-way (avoidant, ambivalent and secure) assessment of attachment type was carried through. None of the studies assessed maternal attachment.

## Discussion

We aimed to systematically review the current status of the literature investigating the early caregiver–infant relationship and attachment in offspring of parents with schizophrenia. We can conclude that although there are data on mother–infant and early care-giving factors, these data are inherently limited by methodological heterogeneity [52]. Most studies included infants aged between 1 and 12 months. Data regarding neonatal characteristics or follow-up of infants beyond 13 months of age were limited. Data from studies of infants in the first 12 months of life suggested evidence of differences in maternal behaviour in schizophrenia compared to controls. As maternal communication deviance is a known risk factor for offspring psychosis [17], this is an important finding. Less consistent differences in infant behaviour compared to controls were found. Specifically, there was evidence of attachment insecurity/avoidance and reduced mutuality of mother–infant interaction in offspring of mothers with schizophrenia compared with controls. These data underscore the possible importance of mother–infant relationship in this clinical group and the need for a conceptual framework to scaffold these and future research studies.

One conceptual framework is attachment theory [53–55]. Attachment theory proposes a developmental model of psychological functioning and affect regulation, emerging from affectional bonds created in the context of close relationships, initially with primary caregivers. Attachment is grounded within the evolutionary need for safety and security [53]. In infancy, attachment behaviour is operationalized through patterns of secure, insecure-avoidant, insecure-ambivalent, and disorganized behaviour [48]. Avoidant and ambivalent behaviour represents strategies to regulate a suboptimal attachment bond, via minimizing or hyperactivating attachment behaviour, respectively, whereas disorganized attachment reflects attachment behaviour characterized by fearful interactions with caregivers. In adulthood, these behavioural patterns are reflected in narrative organization in the Adult Attachment Interview (AAI, [56])—with secure/freely autonomous, insecure dismissing, insecure preoccupied and unresolved with regard to trauma and loss attachment states of mind corresponding to the respective infant patterns.

Attachment research demonstrates that the quality of early caregiver–infant relationship affects developmental risk and resiliency in infants in the general population [57, 58]. Attachment has been associated with affect regulation, stress tolerance and mentalization, which are predictive of risk and resilience during childhood [59]. Attachment insecurity has been found to predict several types of later psychopathology, such as anxiety disorders, depression and antisocial behaviour [60]. In one of the few studies that have followed attachment from infancy to adulthood and linked this to adult psychopathology, disorganized attachment is the strongest single predictor of later psychopathology [61]. In this study, it was also found that attachment disorganization ratings in infancy significantly predicted dissociation in adolescence [62]. Additionally, insecure forms of attachment are more common within psychopathology including psychotic disorders than in normal populations [18, 63]. Based on this evidence, it is reasonable to hypothesize that the quality of early caregiver–infant relationship and attachment could also contribute to risk and resilience in schizophrenia, and could contribute to the diverse diagnostic outcomes in offspring of women with schizophrenia [1, 2] We propose two possible mechanisms for exploring the mother–infant relationship as a context for the transmission of resilience and/or vulnerability to later psychopathology.

### Transmission mechanism 1: quality of mother–infant interaction

Discovery of infant disorganized attachment has led to identification of possible atypical parent–infant interaction patterns. Main and Hesse [64] proposed that frequent interactions with a helplessly frightened, hostile and frightening, or confused caregiver create a relational trap where the infant’s defence system motivates them to flee from the frightened and/or frightening caregivers, while at the same time their attachment system motivates them, influenced by separation fear, to approach them [65]. Thus, the disorganized infant experiences “fright without solution” [64, 66]. This early relational trauma adversely influences the development of the stress-coping system in the infant’s brain [59]. Caregiving behaviours including role-confusion, disorientation and withdrawal have been found to predict infant disorganized attachment [67]. The studies included in this review did not include the measurement of attachment disorganization and thus future studies would benefit from including this.

### Transmission mechanism 2: stress-sensitivity (S–S)

Stress is an important factor in the development of schizophrenia and individuals diagnosed with schizophrenia display increased vulnerability/sensitivity to stress. Empirical evidence supports the view that S–S may not be psychosis specific, but represents a general vulnerability for psychopathology [68]. Thus, a developmental psychopathology approach to schizophrenia has been proposed [69]. It is, therefore, apt to explore to what extent S–S is transmitted from mother to infant in schizophrenia, as this may be a common developmental risk process involved in offspring psychopathological outcomes. S–S can be assessed via psychophysiological studies of cortisol levels and release patterns following stress [70]. Cortisol is a hormone involved in the human stress response. Persons with schizophrenia and at risk of psychosis have higher baseline cortisol levels and exhibit a non-normative cortisol release pattern following stress [68, 71] suggesting increased S–S in schizophrenia. Studies also support an association between severe maternal mental illness and higher infant cortisol levels [72]. The infant–parent relationship is an infant’s most important emotion regulation system in the first 12 months. Early experiences thus shape attachment, thereby influencing regulation of behavioural and physiological responses. Studies of parental care and attachment have identified associations between caregiving environment, attachment classification and infant physiological response to stress. Mothers whose interactions with their infants are most disrupted exhibit most deviation in cortisol levels [73]. Infants with insecure and disorganized attachment classification have elevated cortisol levels during separation in the Strange Situation Procedure (SSP) and disorganized infants showed greatest elevation and slowest return to baseline cortisol levels after SSP [74, 75]. Research [72] found that disorganized infants differed from non-disorganized infants in diurnal cortisol rhythm, displaying a more flattened daily curve. Although not specific to psychosis, these findings suggest links between mother–infant interaction, attachment classification and biological cortisol patterns. Given our findings with regard to mother–infant interaction and attachment classification, we suggest exploration of the role of stress sensitivity as a possible biological mechanism for transmission of resilience/risk from mothers with schizophrenia to their infants.

## Concluding remarks

An important finding of this review is that there is limited evidence of variable quality on the relationship between maternal schizophrenia, the mother–infant relationship and infant development. Therefore, there is an absence of empirical evidence to inform therapeutic interventions and social policy in this area. Given the evidence that parental psychopathology is linked to increased risk of adult psychopathology, there is a clear need for further research exploring the biopsychosocial mechanisms of risk. However, given that almost 50 % of offspring do not develop adult psychopathology, there is an equally pressing need to explore the sources of resilience in this group. Therefore, further research should investigate both sources of resilience and risk in the development of offspring of parents with schizophrenia.

## Limitations

We note that the terminology used to specify outcomes was somewhat varied in identifying studies. This was reflected in the number of additional papers identified by manual search, although this only generated two additional cohorts.

## Electronic supplementary material

Supplementary material 1 (DOCX 104 kb)
