# Supplementary material for: Mother–infant interaction in schizophrenia: transmitting risk or resilience? A systematic review of the literature
Source: Soc Psychiatry Psychiatr Epidemiol. 2015 Oct 10;50(12):1785–98. doi: 10.1007/s00127-015-1127-x (PMC4654744; doi:10.1007/s00127-015-1127-x)
Supplement: Supplementary file 1 — Supplementary material 1 (DOCX 104 kb) [file 127_2015_1127_MOESM1_ESM.docx]

TABLE 3: NEONATAL

**A. Maternal behaviour**

|  | | | | | | **Comparison** | | **Correlates** | |  |
| --- | --- | --- | --- | --- | --- | --- | --- | --- | --- | --- |
| **Cohort & study** | **Infant age** | **Type of interaction** | | **Domain/measures** | **Variables** | **Maternal Schz vs controls** | **Other comparisons** | **Mother**  **correlates** | **Infant correlates** |  |
| **Lund cohort**  [24] | 3 days | Feeding | | Coding  not standard measure  14 maternal variables  3 summary variables | Body contact  Social contact  Tension/uncertainty | Ns  schz < (p=.01)  Ns | *Psychosis vs control*  Ns  Psych < (p=.005)  Psych > (p=.05) | Diagnosis of schz ass. with  disturbed maternal behavior at 3 days |  |  |
| **Bethlem Cohort**  [41] | Neonatal | | Observation  Mother infant interaction | Bethlem Mother Infant interaction Scale (BMIS, Hippwell & Kumar 1996)  Six subscales | Total BMIS score |  | Three group comparison: Schz,depr and bipolar (p=.05-.01)  Schz most disturbed at 2 of 3 assessments | Diagnosis of schz ass. with disturbed maternal behavior in the neonatal period |  |  |

**B. Infant behaviour**

|  | | | | | **Comparison** | | **Correlates** | |  |  |
| --- | --- | --- | --- | --- | --- | --- | --- | --- | --- | --- |
| **Cohort & study** | **Infant age** | **Type of interaction** | **Domain/measures** | **Variables** | **Maternal Schz vs controls** | **Other comparisons** | **Mother**  **correlates** | **Infant correlates** |  | |
| **Lund cohort**  [24] | 3 days | Feeding | Coding not standard measure  7 infant variables  2 summary variables | Social contact  Min. 2 deviations on 4 infant variables | Ns  Ns | Psychosis/control  Psych < (p=.05)  Ns |  | No ass. between maternal schz and infant behavior |  |  |

**C. Mutual interaction**

|  | | | | | | **Comparison** | | **Correlates** | |  |
| --- | --- | --- | --- | --- | --- | --- | --- | --- | --- | --- |
| **Cohort & study** | **Infant age** | | **Type of inter-action** | **Domain/measures** | **Variables** | **Maternal Schz vs controls** | **Other** | **Mother**  **correlates** | **Infant correlates** |  |
| **Lund cohort**  [24] | 3 days | Feeding | | Coding not standard measure  5 interaction variables  1 summary variable | Harmony feeding | Ns | Psychosis/control  Psych < (p=.025) |  | No ass between maternal schz and mutual interaction |  |

Abbrevations:Ns = No Significant findings, Psych = Psychosis

TABLE 4: UP TO 12 MONTHS

**A. Maternal behaviour**

|  | | | | | | | **Comparison** | | **Correlates** | |  |
| --- | --- | --- | --- | --- | --- | --- | --- | --- | --- | --- | --- |
| **Cohort & study** | **Infant age** | **Type of inter-action** | | **Domain/measures** | | **Variables** | **Maternal Schz vs controls** | **Other** | **Mother**  **correlates** | **Infant correlates** |  |
| **Lund cohort**  [25] | 3 & 6 weeks | Feeding  Free play | | Coding not standard measure  19 maternal variables  4 summary variables | | Feeding:  Body contact  Social contact  Tension/uncertainty  Playing:  Body contact  Social contact  Tension/uncertainty | 3w/6w  Ns/Ns  Ns/Schz< (p=.05)  Ns/Schz> (p=.05)  Ns/Schz<(p=.025  Ns/Schz< (p=.05)  Ns/Ns | Psychosis vs. control  3w/6w  Ns/Ns  Ns/Ns  Psych>/Psych>  (p=.005/p=.025)  Ns/Psych<(p=.01)  Psych</Psych<  (p=.05/p=.005)  Psych>/Psych>  (p=.025/p=.025) | Diagnosis of schz ass. with disturbed maternal behavior at 6 but not at 3 weeks |  |  |
| **Lund cohort**  [26] | 3½ & 6 months | Feeding  Free play | | Coding not standard measure  15 maternal variables  3 summary variables | | Feeding:  Body contact  Social contact  Tension/uncertainty  Playing:  Body contact  Social contact  Tension/uncertainty | 3½m/6m  Ns/ns  Ns/ Ns  Ns/ns  Ns/Ns  Ns/Schz<(p=.05)  Ns/Ns | Psychosis vs. control  3½m/6m  Ns/Ns  Ns/Ns  Ns/Ns  Ns/Ns  Psych</Psych<  (p=.025/p=.05)  Ns/Ns | Diagnosis of schz ass. with disturbed maternal behavior at 6 but not at 3½ months |  |  |
| **Lund cohort**  [27] | 1 year | Feeding  Free play | | Coding not standard measure  13 maternal variables  2 summary variables | | Feeding:  Social contact  Tension/uncertainty  Playing:  Social contact  Tension/uncertainty | Ns  Schz>(p=.025)  Ns  Ns | Psychosis vs. control  Ns  Psych>(p=.0005)  Ns  Psych>(p=.005) | Diagnosis of schz ass with disturbed maternal behavior at 12 months |  |  |
| **Lund cohort**  [29] | 3 days,  3&6 weeks  3½, 6 and 12 months | Feeding  Free play (data from 2, 3, 4, 5)  Attachment behavior (data from 6) | | Investigation of antecedents of anxious attachment.  Variables as in 2, 3, 4, 5 and 6 | | Social contact  Body contact  Tension/uncertainty | Psychosis*  Anxious vs. secure  3 days  Ns  Ns  Anxious>(12m, p=.05) | Control*  Anxious vs. secure  3 days  Anxious<(6m, p=.05)  Ns  Ns |  | Anxious attachment in infants of mothers with psychosis correlates with maternal tension/ uncertainty at 12 months |  |
| **Lund cohort**  [32] | 3 days,  3&6 weeks  3½, 6 and 12 months | Feeding  Free play (data from 2, 3, 4 and 5)  Fear of strangers (FOS) (data from 8) | | Investigation of antecedents of fear of strangers  Variables as in 2, 3, 4, 5, 8 | | Social contact  Tension/uncertainty | Psychosis*  FOS- vs. FOS+  Ns  FOS- >  (6w, p=.05/.01  3½ m, p=.05  12m, p=.05) | Controls*  FOS- vs. FOS+  FOS- <  (6w, p=.025  3½m, p=.025 )  Ns | FOS- in infants of mothers with psychosis ass with maternal tension and uncertainty at 6w, 3½ m and12m |  |  |
| **Rochester cohort**  [36] | 4 and 12 months | | 2x2 hours home observati  on | Coding occurrence of 65 types of behaviors at 4 months and 127 at 12 months | | Summary items:  Spontaneous:  Responsive  Proximity:  Smiles  Vocalizes  Happy  Negative  En face  Cuddles  Passive | Schz vs. controls  Schz <(4m)  Ns  Schz <(4m)  Ns  Ns  Ns  Ns  Ns  Ns  Ns | Neurotic dep vs. controls  Dep<(4m)  Ns  Dep<(4m)  Ns  Dep<(4m)  Dep<(4m)  Ns  Ns  Ns  Ns | Diagnosis of schz ass. with only few disturbed maternal behavior at 4 and no at 12 months.  Severity and especially social status significantly correlated with several variables at both 4 and 12 months |  |  |
| **Austin cohort**  [21] | Mean age 16,9 weeks  (1-44 months) | | Unstructured play session  Observation at unit | Infant caregiver behavioral scale  Bethlem mother infant interaction scale (BMIS) | | Response  Stimulation/arousal  Caregiving  Positive affect  Negative affect  Attention  Soothe/comfort  Eye contact  Physical contact  Vocal contact  Mood  General routine  Risk to baby |  | Change during admission **  Ns  Ns  Ns  Ns  Ns  Ns  Ns  X  X  X  X  X  X  (p=.01) | Severity of illness and maternal behavior in interaction significantly associated |  |  |
| **Manchester cohort 1993-95**  [42] | 4 months | | 5 min face to face interaction  (play) | Global Rating Scale of Mother –Infant Interaction (Murray, 1988) | | Warm/pos  Accepting  Responsive  Non-demanding  Sensitive  Non-intrusive behavior  Non-intrusive speech  Non-remote  Non-silent  Happy  Non-flaccid  Absorbed in infant  Relaxed |  | Schz vs affective  Ns  Ns  Schz<  Schz>  Schz<  Schz<  Schz<  Schz<  Schz<  Ns  Schz<  Schz<  Ns | Diagnosis differentiates mother’s behavior. Maternal diagnosis of schz ass with less positive style of interaction |  |  |
| **Manchester cohort 1993-95 + 1996-2000**  [43] | Restricted to 4 months | 5 min face to face interaction  (play) at hospital | | | Global Rating Scale of Mother –Infant Interaction (Murray, 1988) | Warm/pos  Accepting  Responsive  Non-demanding  Sensitive  Non-intrusive behavior  Non-intrusive speech  Non-remote  Non-silent  Happy  Energetic  Absorbed in infant  Relaxed |  | Schz vs. affective  Schz<(p=.05)  Schz<(p=.01)  Schz<(p=.005)  Ns  Schz<(p=.05)  Schz<(p=.05)  Ns  Schz<(p=.005)  Ns  Ns  Ns  Schz<(p=.05)  Ns | Maternal diagnosis differentiates maternal behavior at 4 months Replication of previous results. Additional items where schz was significantly lower: Emotional warmth and acceptance |  |  |
| **Manchester**  **cohort 1996-2000**  [44] | Mean: 15 weeks  (4-60 weeks) | 4 min. face to face interaction  (play) at hospital | | | Modified version of Stanleys et al (2004)  Classification system | Positive response  Negative response  No response |  | Schz vs affective  Schz<(p=.02)  Ns  Ns | Maternal diagnosis differentiates maternal behavior at 4 months.  Schz ass with less positive respons |  |  |
| **Manchester**  **cohort 1996-2000**  [45] | Mean: 16 weeks  (7-60 weeks) | 5 min. face to face unstructured play | | | Coding of several different speech characteristics | Number of utterances  Utterance length  Complete repetition  Cont. of reference  Interrogatives  Imperatives  Declaratives  Infant-focus  Negativity  Songs/rhymes |  | Schz/clinical control group  Ns  Ns  Ns  Ns  Ns  Ns  Ns  Schz<(p=.002)  Ns  Ns | Maternal diagnosis differentiates maternal behavior.  Schz ass with reduced infant-focused speech. |  |  |
| **London cohort**  [46] | Mean: 10.6 weeks at admission  (0-52 weeks) | 5 min  unstructured play | | | Meins & Ferny-hough’s 2006 scheme | Mind-mindedness  Maternal responsiveness  Maternal  intrusiveness | Schz vs. controls  Ns  Ns  Ns | Affective vs. controls  Ns  Ns  p=.001 | Affective group but not schz were more intrusive than controls |  |  |
| **London cohort**  [47] | Mean age: 12.4 weeks  (admission)  20.6 weeks  (discharge) | 3 min unstructured play | | | CARE-index [49] | *Difference in change score (pre-post):*  Maternal sensitive  Maternal unresponsive | Schz vs. depressive  Ns  Ns | Schz vs. mania  Ns  Ns | Significant change in all three diagnostic groups.  No significant effect of diagnosis on the change |  |  |

* no separate data on schz group or comparisons btw psychosis vs. controls in this publication, ** no data on individual item scores, only data on whether change occurs from admission to discharge

**B. Infant behaviour**

|  | | | | | **Comparison** | | **Correlates** | |  |
| --- | --- | --- | --- | --- | --- | --- | --- | --- | --- |
| **Cohort & study** | **Infant age** | **Type of inter-action** | **Domain/measures** | **Variables** | **Maternal Schz vs controls** | **Other** | **Mother**  **correlates** | **Infant correlates** |  |
| **Lund cohort**  [25] | 3 & 6 weeks | Feeding Free play | Coding not stan-dard measure  9 infant variables  1 summary variable | Social contact | 3 w/6 w  Ns/ Ns | Psychosis vs. control  3w/6w  Ns/ns |  | No ass between maternal schz and infant behavior |  |
| **Lund cohort**  [26] | 3½ & 6  months | Feeding Free play | Coding not standard  measure  Variables as above | Feeding:  Social contact  Playing:  Social contact | 3½m/6m  Schz<(p=.05)/Ns  Ns/Ns | Psychosis vs. control  3½m/6m  Psych<(p=.01)/Ns  Ns/Ns |  | Maternal schz associated with reduction of infant’s social contact at 3.5 but not 6 months |  |
| **Lund cohort**  [27] | 1 year | Feeding  Free play | Coding not standard measures  6 infant variables  1 summary variable | Feeding:  Social contact  Playing:  Social contact | Ns  Ns | Psychosis vs. control  Ns  Ns |  | Maternal schz not ass. with infant behavior at 12 months |  |
| **Lund cohort**  [28] | 1 year | Attachment behavior | 3 episodes of separations/reunions | 2 way attachment classification:  Secure vs insecure | Schz vs. control  Schz group more insecure (p = 0.02) | Insecure all groups:  Schz 50%  Nonendog 27%  Cycloid 22%  Affective 17%  Control 18% in | Mothers hospital-lization during first year, but not severity of illness correlated with infant attachment type |  |  |
| **Lund cohort**  [29] | 3 days,  3&6 weeks  3½, 6 and 12 months | Feeding  Free play (data from 2, 3, 4, 5)  Attachment behavior (data from 6) | Investigation of antecedents of anxious attachment.  Variables as in 2, 3, 4, 5 and 6 | Feeding:  Infant social contact  Playing:  Infant social contact | Psychosis*  Anxious/secure  Anxious<(12 m,p=.05)  Anxious<(6m, p=.01) | Controls*  Anxious/secure  Ns  Ns |  | In infants of mothers with psychosis: Reduced infant social contact at 6 months predicts anxious attachment at 12 months  Reduced infant social contact and anxious attachment at 12 months are correlated. |  |
| **Lund cohort**  [30] | 1 year | Observer/stranger arriving at infant’s home | Coding of standardized procedure (Schaffer and Emerson, 1964) | Fear of strangers | Schz vs. controls  Schz< (p=.04) | Psychosis vs. controls  Psych< (p=.025) | Fear of strangers unrelated to mothers hospitalization during first year and severity of illness | Infants of mothers with schz display reduced fear of strangers. Fear of strangers unrelated to infant gender |  |
| **Lund cohort**  [31] | 1 year | Exploratory behavior | Coding of exploratory behavior during the Strange Situation Procedure, Näslund shortened version | Exploration | Schz vs. controls  Ns | Psychosis vs. controls  Ns |  | Infants of mothers with schz do not display deviations in exploratory behavior compared to normal controls |  |
| **Lund cohort**  [32] | 3 days,  3&6 weeks  3½, 6 and 12 months | Feeding  Free play (data from 2, 3, 4 and 5)  Fear of strangers (FOS) (data from 8) | Investigation of antecedents of fear of strangers  Variables as 2, 3 4, 5, 8 | Child social contact | Psychosis*  FOS- vs. FOS+  Ns | Controls*  FOS- vs. FOS+  FOS- <  (6w, p=.01) |  | Fear of strangers in infants of mothers with psychosis unrelated to infant behavior |  |
| **Boston Cohort**  [40] | Mean age 12, 5 months | Attachment behavior | Strange Situation Procedure, Näslund shortened version  SSP full version | 2 ways: anxious vs secure  2 ways: anxious vs. secure  3 ways: aviodant, ambivalent, secure | Schz vs. controls  Anxious/secure  Schz> (p=.05)    Schz> (p=.005)  Schz more avoidant (p=.05) | Dep vs controls  Anxious/secure  Dep> (p=.01)  Dep more ambivalent (p=.05) |  | Maternal diagnosis of schz ass with insecure infant attachment at 1 year |  |
| **Rochester Cohort**  [36] | 4 and 12 months | 2x2 hours home observation  Abbreviated Strange Situation Procedure | Coding occurrence of 65 types of mother and infant behaviors at 4 months and 127 at 12 months | Summary items:  Spontaneous  Responsive  Happy  Object-orientation  Cries  Negative  Clings  Whimpers  Movement  Plays  Attachment | Ns  Ns  Ns  Ns  Ns  Ns  Ns  Ns  Ns  Ns  Ns | Neurotic dep vs. controls  Ns  Ns  Ns  Ns  Ns  Ns  Ns  Ns  Ns  Ns  Ns | Maternal diagnosis unrelated to infant behavior at 4 and 12 months.  Maternal social status significant for several infant variables at 12 but not at 4 months. |  |  |
| **Austin cohort**  [21] | Mean age 16.9 weeks  (1-44 weeks) | Unstructured play session  Observation at unit | Infant caregiver behavioral scale  Bethlem mother infant interaction scale (BMIS) | Clarity of cues  Exploration  Smile/excite  Fuss/cry  Attention to other children  Attention to caregiver  Aggression  Alertness |  | Change during admission  Ns  X (p=.05)  X (p=.05)  Ns  Ns  Ns  Ns  X (p=.05) |  | No infant correlates |  |
| **Manchester Cohort 1993-1995**  [42] | 4 months | 5 min face to face interaction (play) at hospital | Global Rating Scale of Mother –Infant Interaction (Murray, 1988) | Attentive to mother  Active communic.  Pos. vocalization  Engaged with env.  Lively  Happy  Non-fretful |  | Schz vs affective  Schz<  Ns  Ns  Ns  Ns  Ns  Ns |  | Maternal diagnosis differentiates infant behavior at 4 months  Maternal diagnosis of schz ass with infants reduced attention to mother (more avoidant) |  |
| **Manchester cohort 1993-95 + 1996-2000**  [43] | Restricted to 4 months | 5 min face to face interaction  (unstructured play) at hospital | Global Rating Scale of Mother –Infant Interaction (Murray, 1988) | Attentive to mother  Active communic.  Pos. vocalization  Engaged with env.  Lively  Happy  Non-fretful |  | Schz vs affective  Schz<(p=.005)  Schz<(p=.05)  Ns  Schz<(p=.01)  Schz<(p=.05)  Ns  Ns | Maternal diagnosis differentiates infant behavior at 4 months. | Infant attentiveness ass with maternal sensitivity/responsiveness. Schz infants less attentive to mother (more avoidant), less engaged with environment, less lively. |  |
| **Manchester**  **cohort 1996-2000**  [44] | Mean: 15 weeks  (4-60 weeks)  20 male  25 female | 5 min face to face interac-tion(unstruc-tured play) at hospital | Modified version of Stanleys et al (2004)  Classification system | Positive response  Negative response  Neutral/ambiguous |  | Schz vs affective  Ns  Ns  Ns |  | Maternal diagnosis did not differentiate infant behavior at 4 months |  |
| **London cohort**  [46] | Mean: 10.6 weeks at admission  0-52 weeks | 5 min unstructured play |  | Change in gaze  Gazes to mother |  | Between all groups  Ns  Ns |  |  |  |
| **London cohort**  [47] | Mean age: 12.4 weeks  (admission)  20.6 weeks  (discharge) | 3 min unstructured play | CARE-index [48] | *Difference in change score (pre-post):*  Infant cooperative  Infant passive | Schz vs. depression  Ns  Ns | Schz vs. mania  Ns  Ns |  | Significant change in all three diagnostic groups.  No significant effect of diagnosis on the change |  |

**C. Mutual interaction**

|  | | | | | | **Comparison** | | **Correlates** | |  |
| --- | --- | --- | --- | --- | --- | --- | --- | --- | --- | --- |
| **Cohort & study** | **Infant age** | **Type of inter-action** | **Domain/measures** | | **Variables** | **Maternal Schz vs controls** | **Other** | **Mother**  **correlates** | **Infant correlates** |  |
| **Lund cohort**  [25] | 3 & 6 weeks | Feeding  Free play | | Coding not standard  measure  5 interaction  variables  2 summary variables | Harmony feeding  Reciprocal behaviors | 3 w/6 w  Ns/Schz<(p=.01)  Ns/Ns | Psychosis vs. control  3 w/6 w  Psych</Psych<  (p=.05/p=.01)  Ns/Ns |  | Maternal diagnosis of schz ass. with disturbed mutual interaction at 6 but not 3 weeks |  |
| **Lund cohort**  [26] | 3½ & 6  months | Feeding  Free play | | Coding not standard measure  Variables as above | Harmony feeding  Feeding:  Reciprocal behaviors  Playing:  Reciprocal behaviors | 3 ½m/6 m  Ns/ Ns  Ns/ Ns  Ns/Ns | Psychosis vs. control  3 ½m/6 m  Ns/ns  Psych<(p=.05)/Ns  Psych</Psych<  (p=.05/p=.05) |  | Maternal schz not ass. with disturbed reciprocity at 3½ and 6 months |  |
| **Lund cohort**  [27] | 1 year | Feeding  Free play | | Coding not standard measures  8 interaction variables  2 summary variables | Harmony feeding  Feeding:  Reciprocal behaviors  Playing:  Reciprocal behaviors | Ns  Ns  Ns | Psychosis vs. control  Ns  Ns  Ns | Maternal schz not ass. with reciprocal behaviors at 1 year |  |  |
| **Lund cohort**  [29] | 3 days,  3&6 weeks  3½, 6 and 12 months | Feeding  Free play  (data from 2, 3, 4 and 5)  Attachment behavior (data from 6) | | Investigation of antecedents of anxious attachment.  Variables as in 2, 3, 4, 5 and 6 | Harmony feeding  Reciprocal behaviors | Psychosis*  Anxious/secure  Anxious<  (3w, p=.01;  12m,p=.005 )  Anxious<(6m) | Controls*  Anxious/secure  Anxious<  (6w, p=.025;  6m, p=.05  12m, p=.025)  Ns | Anxious attachment in infants of mothers with psychosis predicted by disturbances in recipro-city at 3 weeks and 6 months and correlated with distur-bed reciprocity at 12 months |  |  |
| **Lund cohort**  [32] | 3 days,  3&6 weeks  3½, 6 and 12 months | Feeding  Free play  (data from 2, 3, 4, 5)  Fear of strangers (FOS) (data from 8) | | Investigation of antecedents of fear of strangers  Variables as in 2, 3, 4, 5, 8 | Harmony feeding  Reciprocal behaviors | Psychosis*  FOS- vs. FOS+  FOS- <  (6m, p=.01)  Ns | Controls*  FOS- vs. FOS+  Ns  FOS- <  (6w, p=.025  6m, p=.005) |  | Absent fear of strangers in infants of mothers with psychosis associated with less harmony in feeding at 6 months |  |
| **Austin cohort**  [21] | Mean age 16.9 weeks (1-44 months) | Unstructured play session  Observation at unit | | Infant caregiver behavioral scale  Bethlem mother infant interaction scale (BMIS) | Mutual attention  Reciprocity/synchronicity  Intensity of interaction |  | Change during admission  X  X  X  (p=.05) |  |  |  |
| **Manchester Cohort 1993-95**  [42] | App. 4 months | 5 min face to face interaction  (play) at hospital | | Global Rating Scale of Mother –Infant Interaction (Murray, 1988) | Smooth/easy  Fun  Mutually satisfying  Much engagement  Excited engagement |  | Schz vs affective  Ns  Schz<  Schz<  Schz<  Schz< |  | Maternal diagnosis differentiates mutual interaction. Maternal diagnosis of schz ass with less mutually satisfying, less engaged interaction |  |
| **Manchester cohort 1993-95 + 1996-2000**  [43] | Restricted to 4 months | 5 min face to face interaction  (play) at hospital | | Global Rating Scale of Mother –Infant Interaction (Murray, 1988) | Smooth/easy  Fun  Mutually satisfying  Amount of engagement  Excited engagement |  | Schz vs affective  Schz<(p=.05)  Schz<(p=.05)  Schz<(p=.01)  Schz<(p=.005)  Schz<(p=.05) |  | Maternal diagnosis differentiates mutual interaction at 4 months. All single items significantly different. Interactions less smooth and easy. |  |

Abbrevations: Ns = No Significant findings, Psych = Psychosis

TABLE 5. 13-36 MONTHS

**A. Maternal behaviour**

|  | | | | | **Comparison** | | **Correlates** | |  |
| --- | --- | --- | --- | --- | --- | --- | --- | --- | --- |
| **Cohort & study** | **Infant age** | **Type of inter-action** | **Domain/measures** | **Variables** | **Maternal Schz vs controls** | **Other** | **Mother**  **correlates** | **Infant correlates** |  |
| **Pittsburg cohort**  [39] | Mean: 14.2 months | Feeding in laboratory | Coding not standard measure  7 maternal variables | Controls  Offers tangible items  Pos./aff. behavior  Plays  Neutral behavior  Neg./angry behavior  Attends to other than baby | Ns  Ns  Ns  Ns  Ns  Ns  Ns |  | Diagnosis of schz does not differentiate maternal behavior |  |  |
| **Emory cohort**  [20] | 0-5 years  (mean: 2 years) | 5 min. semistructured play session in laboratory  Observation in home | Coding with Mother’s Project Rating Scale of Mother-Child Interaction (Clark et al., 1980)  HOME inventory | Affectional involvement and responsiveness  Tenseness  Anger and hostility  Child rearing environment , total  Maternal responsiveness  Play stimulation  Variety of stimulation  Avoidance of punishment and discipline | Schz vs. Control  Ni  Ni  Ni  Schz<(T1,2,3)  Schz<(T1,2,3)  (p=.006, .002, .03)  Schz<(T1,2,3)  (p=.03, .04, .06)  Schz<(T1, p=.003)  Ni | Dep vs. control  Ni  Ni  Ni  Dep<(T2)  Ni  Ni  Ni  Ni | Diagnosis of schz consistently ass with less responsiveness towards infants and less stimulation of infants | Diagnosis of schz ass with lower infant IQ scores at first testing  Maternal affective involvement ass with infant IQ and infant social behavior for total sample, not schz alone. |  |
| **Emory cohort**  [37] | Assessment at referral (infant < 5 years old) | Semistructured 5 min. play session in laboratory  and  Observation in home | Coding with Mother’s Project Rating Scale of Mother-Child Interaction (Clark et al., 1980)  31 maternal items  HOME inventory | Affectional involvement and responsiveness  Tenseness  Anger and hostility  Child rearing environment, total  Maternal responsiveness  Play stimulation  Variety of stimulation  Avoidance of punishment and discipline | Schz vs. control  Schz<(p=.001)  Ns  Schz<(p=.05)  Schz<(p=.04)  Schz<(p=.006)  Schz<(p=.03)  Schz<(p=.003)  Schz< | Depressive vs. control  Depr<(p=.05)  Ns  Ns  Depr<(p=.05)  Depr<  Depr<  Ns  Depr<(p=.02) | Diagnosis of schz ass with less affectional involvement and responsiveness with children , and providing a poorer child environment |  |  |

**B. Infant behaviour**

|  | | | | | **Comparison** | | **Correlates** | |  |
| --- | --- | --- | --- | --- | --- | --- | --- | --- | --- |
| **Cohort & study** | **Infant age** | **Type of inter-action** | **Domain/measures** | **Variables** | **Maternal Schz vs controls** | **Other** | **Mother**  **correlates** | **Infant correlates** |  |
| **Pittsburgh cohort**  [39] | Mean: 14.2 months  Gender: Ni | Feeding in laboratory | Coding not standard measure¨  7 infant variables | Fusses/cries  Seeks or takes  Approach/compliance  Plays  Physiological events  Neg./angry behavior  Attends to other than mother | Ns  Ns  Ns  Ns  Ns  Ns  Ns |  | Maternal diagnosis not associated with deviant infant behavior |  |  |
| **Emory cohort**  [20] | 0-5 years  3 assessment points, each one year apart | 5 min. play session in laboratory  30 minutes mother-child interaction in home | Coding with Mother’s Project Rating Scale of Mother-Child Interaction (Clark et al., 1980)  Child social behavior coded with  Burton White’s (1978) observational procedure and checklist scoring system | Affect  Disposition (angry,  hostile+anxiety)  Activity level  Behavior (communicative competence)  Getting the attention of mother  Expressing affection or mild annoyance  Using mother as resource  Pride in achievement  Role play | Schz vs control  TIME 1  Schz<(T1,2)  (p=.02, .04)  Schz<(T2)  (p=.05)  Schz> (T1)  (p=.04)  Schz<(T1)  (p=.01)  Ns  Schz<(T1)  (p=.02)  Schz<(T1)  (p=.02)  Ns  Schz<(T1)  (p=.03) | Dep vs control  TIME 1  Dep<T2  (p=.04)  Ns  Ns  Ns  Ns  Ns  Dep<(T1)  (p=.02)  Dep<(T3)  (p=.01)  Dep<(T1,3)  (p=.03, .02) | **Infants of mothers with diagnosis of schz not consistently associated with deviant infant behavior** | Infants of mothers with schz not consistently associated with deviant infant behavior (primarily at first assessment).  Psychiatric outcome in offspring equally likely for schz and depr mothers at each of the three testings |  |
| **Emory cohort**  [37] | Assessment at referral (infant < 5 years old) | Semistructured 5 min. play session in laboratory  and  30 minutes mother-child interaction in home | Coding with Mother’s Project Rating Scale of Mother-Child Interaction (Clark et al., 1980)  15 infant items  Child social behavior coded with  Burton White’s (1978) observational procedure and checklist scoring system | Affect  Disposition  Activity level  Behavior  Getting the attention of mother  Expressing affection or mild annoyance to mother when appropriate  Using mother as resource  Pride in achievement  Role play | Ni  Ni  Ni  Ni  Ni on diagnosis | Ni  Ni  Ni  Ni |  | Maternal affectional involvement and responsiveness ass with child IQ (p<0.2) and child’s role play (p<0.009) |  |

**C. Mutual interaction**

|  | | | | | | **Comparison** | | **Correlates** | |  |
| --- | --- | --- | --- | --- | --- | --- | --- | --- | --- | --- |
| **Cohort & study** | **Infant age** | | **Type of inter-action** | **Domain/measures** | **Variables** | **Maternal Schz vs controls** | **Other** | **Mother**  **correlates** | **Infant correlates** |  |
| **Pittsburgh cohort**  [39] | Mean: 14,2 months  Gender: Ni | Feeding in laboratory | | Coding not standard measures  Contingencies derived through sequential analysis of video recording | Mother behavior following presence of baby behavior  Mother behavior following absence of baby behavior  Baby behavior following presence of mother behavior  Baby behavior following absence of mother behavior | Schz> (p=.001)  Ns  Schz> (p=.001)  Schz< (p=.01) |  | Education not correlated with interaction  Less adequate care correlated with high mother following baby behavior (p=.001), | Number of developmental problems correlated with high mother following baby behavior |  |
| **Emory cohort**  [37] | Assessment at referral (infant < 5 years old) | Semistructured 5 min. play session in laboratory  and | | Coding with Mother’s Project Rating Scale of Mother-Child Interaction (Clark et al., 1980 | Affective quality  (anger and hostility) | Schz< (p=.05) | Dep vs. controls  Ns | Diagnosis of schz associated with interactions characterized by less anger and hostility |  |  |

Abbrevations: Ns = No significant findings, Ni= Not indicated, Psych = Psychosis

TABLE 6. ABOVE 36 MONTHS (No data on correlates between maternal behavior or mutual interaction and infant outcome above 36 months)

**B. Infant behaviour**

|  | | | | | **Comparison** | | **Correlates** | |  |
| --- | --- | --- | --- | --- | --- | --- | --- | --- | --- |
| **Cohort & study** | **Infant age** | **Type of interaction** | **Domain/measures** | **Variables** | **Maternal Schz vs controls** | **Other** | **Mother**  **correlates** | **Infant correlates** |  |
| **Lund cohort**  [34] | 1 and 6 years | Attachment behaviour | SSP shortened version  Observation procedure at home  Childrens Global Assessment Scale (CGAS) | Secure /anxious attachment  Fear of strangers  Global Psychoapthology | Schz vs. control  Ni  Ns  Schz>  (82%, p=.002) | Psychosis vs. control  Trend ass.  Ns  Psych>  (50%, p=.004) |  | Attachment at 1 year showed (only) a trend association with psycho-pathology at 6 year |  |

Abbrevations: Ns = No significant findings, Ni= Not indicated, Psych = Psychosis
